# Psychological, situational and application-related determinants of the intention to self-test: a factorial survey among students

**DOI:** 10.1186/s12913-017-2394-x

**Published:** 2017-07-10

**Authors:** Pinar Kuecuekbalaban, Tim Rostalski, Silke Schmidt, Holger Muehlan

**Affiliations:** Department Health & Prevention, Ernst-Moritz-Arndt-University Greifswald, Robert-Blum-Str.13, D-17487 Greifswald, Germany

**Keywords:** Self-testing, Self-diagnosis, Self-management, Health behaviour (theories), Factorial survey

## Abstract

**Background:**

The Internet enables an unprecedented opportunity to access a broad range of self-tests (e.g. testing for HIV, cancer, hepatitis B/C), which can be conducted by lay consumers without the help of a health professional. However, there is only little knowledge about the determinants of the use of self-tests. Thus, the aims of this study were (1) to *experimentally* investigate the impact of situational and application-related characteristics on the intention to use a self-test (ST), compared to being tested by a health professional at home (HPH) or at a doctor’s office (HPD), (2) to examine the applicability of social-cognitive health behaviour theories on self-testing, and (3) to explore the advantages of integrating technological affinity into social-cognitive health behaviour models to predict self-testing.

**Methods:**

In a factorial survey, 1248 vignettes were rated by 208 students. The core concepts of social-cognitive health behaviour theories, technological affinity, and different situational and application-related characteristics were investigated.

**Results:**

Intention to ST was only predicted by the medical expertise of the tested person, while HPH and HPD were also associated with the application purpose of the test and the presence of an emotionally supporting person. Perceived severity and outcome-expectancy significantly predicted intention to self-test. Technological enthusiastic people had a higher intention to use a self-test.

**Conclusions:**

Intention to ST, HPH and HPD were predicted by different situational and application-related characteristics. Social-cognitive health behaviour theories can be applied to predict self-testing and do not need to be extended by technological affinity.

**Electronic supplementary material:**

The online version of this article (doi:10.1186/s12913-017-2394-x) contains supplementary material, which is available to authorized users.

## Background

A broad range of self-tests (testing for e.g. HIV, anaemia, Chlamydia) has become available to the European public via the Internet [1, 2]. Self-tests can be defined as tests on body materials (e.g. blood, urine, faeces, saliva) that are initiated by consumers to diagnose a particular disorder or risk factor, and that are conducted without the involvement of a health professional [3]. Consumer autonomy, self-management, empowerment, privacy protection and convenience due to the absence of a doctor are mentioned as some of the advantages of self-testing (e.g. [2, 4]). On the other hand, disadvantages include concerns about the safety of self-testing, the very low sensitivities displayed by some self-tests [5], the risk of false reassurance in the case of false-negative test results, and the risk of anxiety in the case of true-or false-positive test results as well as unnecessary medical investigations in the case of false-positive results [6–8]. Furthermore, the instruction leaflets of self-tests have been found to be limited on information regarding reliability, follow-up behaviour, and the target group of the test [9].

Despite the potential risks of self-testing, results of surveys from the Netherlands showed that 16% respondents of a Dutch Internet survey confirmed the use of at least one self-test, while in the UK 13% of the British participants of a written survey had used a self-test at least once [2, 10]. Similarly, results of a representative survey in Germany of more than 2500 participants showed that 8% of the German population had used at least one self-test, and about one third of these had used different self-tests [11]. Given the current shortage of physicians in Germany [12], especially in rural areas [13], the need for and use of self-tests could increase in the future. This assumption is furthermore supported by the results of interviews with experts in the development of innovative medical diagnostic devices, who expect a further technological breakthrough of diagnostic devices for end-users in the coming 10 years [14].

The usage of self-tests is embedded in the topics of screening behaviour and disease prevention behaviour in health psychology. Beyond that, however, self-testing presents a new field of application for the validation of common health belief models, which traditionally investigated behaviours such as smoking, alcohol consumption or eating. Although some studies have investigated the psychological determinants of self-testing (e.g. [3, 8, 15, 16]), none of these has considered the technological component of self-testing, for example by integrating technological affinity as an additional predictor for the decision to use a self-test. Thus, this study investigated the role of *technological affinity* – defined as the attraction to technological devices [17] – when included as an additional predictor into the core concepts of health behaviour theories. It was proposed that the greater the enthusiasm, positive attitude and competence towards technological devices, the greater the intention to use a self-test.

Furthermore, three factors were identified, which in accordance with Hahn and von Lengerke [18] represent the core concepts of health behaviour theories: (a) *risk perception*, (b) *self-efficacy*, and (c) *outcome expectancy* (see Additional file 1: Table S1). *Risk perception* is a central variable in e.g. the Health Belief Model (HBM), the Protection Motivation Theory (PMT), and the Theory of Planned Behavior (TPB) [19–22]. In the Health Action Process Approach (HAPA), *risk perception* is composed of (a) the individual’s belief of the seriousness of a certain disease/condition (*perceived severity*), and (b) the individual’s belief of the chance of contracting a certain disease/condition (*perceived susceptibility*) [23]. Additionally, a positive correlation between *technological affinity*, which is understood as a person’s attraction to technological devices [17], and the intention to use a self-test was proposed.

Moreover, the available research has predominantly investigated the psychological determinants of self-testing by conducting interviews or surveys. To the best of our knowledge, no research has been conducted so far to *experimentally* investigate the impact of situational and application-related characteristics of a test situation on the intention to use a test, such as the test result being displayed immediately on the device, versus the sample being analysed in a laboratory and the result communicated in written form.

Thus, this study had three objectives. First, to *experimentally* investigate the impact of situational and application-related characteristics of the test situation on the intention to use a self-test (ST) versus being tested by a health professional at home (HPH), or in a doctor’s office/hospital (HPD). The HPH setting represents an intermediate scenario between the ST and HPD settings. Second, to investigate whether the core concepts of health behaviour theories can predict the intention to use a self-test. And third, to examine whether taking *technological affinity* into account as an additional predictor improves the predictive value of the core concepts of health behaviour theories.

## Methods

### Methodological implementation – Factorial survey

To experimentally investigate the impact of situational and application-related factors, a factorial survey, also called a *vignette analysis*, was conducted [24]. Vignettes are fictive descriptions of a situation or person, constructed by systematically combining all values of factors (predictors) which are believed to influence a judgment being studied by a rating task (criterion, [24–26]). Vignette analyses are a common method in sociology. In a review of 106 articles from 1982 to 2006, the factorial surveys were most frequently used to measure normative judgments (*n* = 62) and positive beliefs (*n* = 26), they were also often used to examine own (intended) actions (*n* = 22) [26]. The latter is the aim of this survey.

The method of a vignette analysis has a number of advantages for the study presented here. First, vignette analysis is particularly appropriate for investigating context- and condition-related research questions. This is because the respondents, rather than being confronted with abstract values, are presented with concrete and detailed descriptions of a situation, where several different characteristics are systematically varied [24, 26]. This is especially relevant for the current research question regarding the impact of situational and application-related characteristics of self-testing. Second, in a factorial survey, the principles of an experiment are combined with a social survey [24, 26]. While the first is associated with a high internal validity, the latter is distinguished by a high external validity. Third, a factorial survey is less subject to social desirability bias than conventional surveys are, because the respondents are not likely to be fully aware of all systematically varied characteristics of a situation, and/or they can be forced to judge two socially equally undesired statements at the same time [24, 27]. Finally, factorial surveys are particularly appropriate when researchers want to study actual determinants and combinations of determinants of human judgments, because persons might not be aware of the influences of certain factors on their judgments, and therefore they might not be capable of explicating such influences [28].

### Measures – Situational and application-related characteristics

The final set of situational and application-related characteristics of a test situation was identified using four approaches. First, multiple case histories were developed to cover a broad range of varying kinds of diagnostic test situations within the framework of the research consortium DIA-LOC (http://m-health.psychologie.uni-greifswald.de/dialoc/index.html) [29]. This way, the importance of the factors *application purpose* and *seriousness of the situation* was identified, and their values were specified. Second, the literature was reviewed in terms of the application-related characteristics of innovative in-vitro diagnostic devices which can be used outside a laboratory, the so-called lab-on-a-chip systems (LOCs). LOCs are designed for a broad spectrum of *application purposes*, such as risk assessment, pre-symptomatic diagnostics, early detection of a disease, and therapy control [30]. Third, an ontology for LOCs was developed within the framework of our research consortium, to further distinguish between the factors *application purpose* and *setting of the test* [31]. And finally, the relevance of the previously identified factors was evaluated in a survey by (a) experts in LOC research and development, (b) experts in health technology assessment, and (c) our interdisciplinary research group. *Analysis and feedback of the test results*, *medical expertise of the tested person,* and *emotional support* were identified as three additional factors.

Table 1 gives an overview of the six factors mentioned above with their several values and frequency of occurrence in the factorial survey. Since the factor *setting of the test* was used as a grouping variable, 337 rated vignettes were related to a ST, 478 to a HPH, and 433 to a HPD situation. For example, the *application purpose* monitoring of a disease/condition occurred in 16.6% of the vignettes of the ST, in 15.3% of the HPH, and in 18.2% of the HPD group.

**Table 1:** Overview of situational and application-related vignette dimensions

		ST		HPH		HPD	
		n	%	n	%	n	%
Application purpose							
1. Risk assessment		43	12.8	98	20.5	67	15,5
2. Early detection of a disease		57	16.9	65	13.6	86	19.9
3. Clinical diagnostics		66	19.6	100	20.9	42	9.7
4. Therapy diagnostics		34	10.1	66	13.8	108	24.9
5. Drug effect		81	24.0	76	15.9	51	11.8
6. Monitoring		56	16.6	73	15.3	79	18.2
Seriousness of the situation							
1. Acute and life-threatening		115	34.1	82	17.2	113	26.1
2. Acute, but not life-threatening		70	20.8	138	28.9	98	22.6
3. Chronic, slowly advancing and life-threatening		59	17.5	91	19.0	89	20.6
4. Chronic, but not life-threatening		93	27.6	167	34.9	133	30.7
Setting of the test							
1. Independently at home without the presence of a health professional		337	27.0	0	0	0	0
2. Tested by a health professional at home		0	0	478	38.3	0	0
3. Tested by a health professional in the doctor’s office/hospital		0	0	0	0	433	34.7
Analysis and feedback							
1. Analysed automatically, and the result is displayed immediately		56	16.6	105	22.0	94	21.7
2. Transmitted automatically and only a conspicuous result is communicated by a health professional		75	22.3	0	0	0	0
3. Transmitted automatically and the result is communicated by a health professional		125	37.1	0	0	0	0
4. Transmitted automatically and the result is communicated in written form		81	24.0	0	0	0	0
5. Analysed in a laboratory and only a conspicuous result is communicated by a health professional		0	0	167	34.9	130	30.0
6. Analysed in a laboratory and the result is communicated by a health professional		0	0	105	22.0	82	18.9
7. Analysed in a laboratory and the result is communicated in written form		0	0	101	21.1	127	29.3
Medical expertise of the tested person							
1. No		112	33.2	126	26.4	148	34.2
2. Unprofessional		135	40.1	167	34.9	130	30.0
3. Professional		90	26.7	185	38.7	155	35.8
Emotional support							
1. Not present		120	35.6	145	30.3	168	38.8
2. Potentially available		119	35.3	149	31.2	131	30.3
3. Personally present		98	29.1	184	38.5	134	30.9

**Fig. 1:**
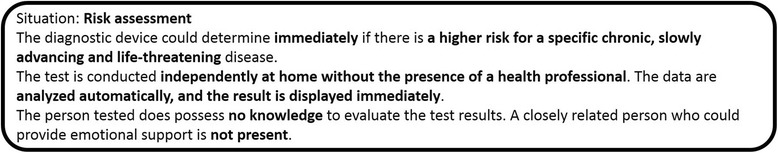
Example of a vignette

Since the given vignette universe of the six situational and application-related factors (Cartesian product: 6 × 4 × 3 × 7 × 3 × 3 = 4536) was far too large to judge all possible combinations, a sample of vignettes (decks of vignettes/subsets) was drawn, and the respondents were presented different selections of the reduced vignette universe. This is a common method in factorial surveys [26, 32]. The reduced vignette universe was selected by a conditional random sampling. To achieve a balanced ratio of the six values of the factor *application purpose*, 30 vignettes of each value were drawn randomly without replacement, so that the vignette population was reduced to 180 vignettes. Implausible combinations of factor values were deleted before the selection of the vignettes. Figure 1 shows a single vignette of the situation risk assessment. To avoid fatigue and a high number of dropouts, six vignettes were presented to each participant – one vignette of every value of the factor application purpose.

### Measures – Psychological characteristics

Before presenting the vignettes, the socio-demographic characteristics and psychological predictors self-efficacy, perceived susceptibility, and technological affinity were assessed once before the vignettes were presented. The other psychological variables *perceived severity* and *outcome expectancy* were presented after each of the six vignettes, because the participants needed to imagine themselves into the vignette scenario to be able to assess these two predictors. The Additional file 1: Table S1 provides an overview of the psychological predictors, their conceptual definitions, items, and answering options. *Self-efficacy,* which is defined as the individual’s confidence in one’s capability to successfully perform a certain action, was measured with the well-established General Self-Efficacy scale (GSE, 10 items, *M* = 28.39, *SD* = 4.11, Cronbach’s α = .85, [33]). According to Karrer, Glaser and Clemens [17], *technological affinity* is defined as a personality trait which is manifested in a positive attitude, enthusiasm, and trust in electronic devices (e.g. mobile phones, computers, personal digital assistants). It was measured by applying three scales, which are all included in the German Technological Affinity Assessment (TA-EG, [17]). Subscale scores were computed and the means calculated for the subscales enthusiasm (5 items, *M* = 14.46, *SD* = 3.32, Cronbach’s α = .83), positive attitude (5 items, *M* = 17.62, *SD* = 2.80, Cronbach’s α = .69), and competence towards electronic devices (4 items, *M* = 14.40, *SD* = 2.87, Cronbach’s α = .74). To assess *perceived susceptibility*, the individual’s belief of the chance of contracting a certain disease/condition, the following item was adopted from the ‘Berlin Risk Appraisal and Health Motivation Study’ (BRAHMS, [34]): the question ‘How high do you rate the probability that at some time you will get ...’, and its response format ‘very unlikely’ to ‘very likely’. While the BRAHMS project investigated the *perceived susceptibility* for specific diseases (e.g. risk of heart attack), in this survey, the items were adopted to fit to the vignette factor ‘seriousness of a situation’. Thus, the *perceived susceptibility* of contracting a non-specific ‘acute vs. chronic, non-life-threatening vs. life-threatening’ disease was investigated. The four adjusted *perceived susceptibility* items were summed up to yield a final composite score (4 items, *M* = 11.96, *SD* = 3.73, Cronbach’s α = .84).

*Perceived severity,* the individual’s belief of the seriousness of a certain disease/condition, (1 item, *M* = 56.88, *SD* = 30.03), and *outcome expectancy*, the individual’s weighting of the positive and negative consequences of acting and not acting, (1 item, *M* = 59.53, *SD* = 23.18), were also adopted from BRAHMS. They were adjusted to fit into the fictive vignette scenarios, for example by adding the term ‘… if the test depicted in the above situation were not conducted’, to ensure that the participants imagine themselves in the presented situation before assessing the above two predictors. Furthermore, the criterion *intention to use the test* was measured after every vignette with the question ‘Would you make use of a test that is conducted as described in the situation above?’ with a response scale from 1 = ‘certainly not’, to 100 = ‘most certainly’ (1-item, *M* = 63.32, *SD* = 29.37).

### Statistical analyses

The descriptive analyses were conducted using IBM SPSS Statistics 22.0 [35]. Because each participant judged more than one vignette, the vignettes were nested within a person, violating a primary assumption of linear regression analysis, the independence of error values [26]. To solve this problem, multilevel regression models were calculated, allowing for modelling the within (vignette characteristics) and between (respondent characteristics) variance. Four models of increasing complexity were applied with the mixed modelling tool (xtmixed) of the STATA software, using the maximum likelihood estimates (mle option) [36, 37]. The first model was a constant-only empty model without any additional predictors (RIO model). The second model examined the impact of the situational and application-related predictors which were operationalised in the vignettes (RI_V_all_ model). The third model investigated the impact of additional *technological affinity* (RI_V_all__P_TA_), and in the fourth model, the health psychological factors were added as predictors (RI_V_all__P_all_ model). Categorial variables were dummy-coded. Metric variables were centred on the grand mean, prior to entering them into the models.

For each model, the deviances, which indicate how well the models fit the data and which are defined as −2 times the log-likelihood, were calculated ([38], p. 47). Subsequently, using a log rank test, the more complex models were compared to the simpler models regarding their model fit. As a statistic analogous to the multiple *R* ^*2*^ from ordinary multiple regression analyses, the reduction of the residual error variances in a sequence of models was examined ([38], p. 69–71). In particular, the reduction of the *error variance within* was calculated in two consecutive models to examine the impact of the situational and application-related factors, and the reduction of the *error variance between* was calculated in two consecutive models to investigate the impact of *technological affinity* and the health psychological predictors. Each model was separately calculated for the three settings ST, HPH and HPD, by using the vignette factor *setting of the test* as a grouping variable.

To facilitate the interpretation of the results, the values of the vignette factors were recoded in such a way that their total mean in dependence of the criterion was in ascending order (see Additional file 2: Table S2). The confounding structure of the parameter estimates was investigated by the alias() function in the statistical programming language R [39]. This test showed that none of the estimated parameters in our model was confounded with any interaction effect.

## Results

### Respondent characteristics

A random sample of university students were approached via an email distribution list and an online survey was conducted in Germany. Initially, 566 participants started the survey, but there was a remarkable dropout before the vignettes were presented, which implies that a huge proportion of participants decided to decline the survey after they were more familiar with the subject of the study. From those 319 participants who already responded to the first vignette 239 participants completed all six vignettes. Thus, nearly 75% of all participants, who actively decided to answer the vignettes, had finished this section. Finally, we excluded 31 cases due to potential response bias. In the end, 1248 vignettes, which were rated by 208 students, were included in the analyses. The majority of respondents were female (76.4%). The age of the participants varied between 18 and 52 years (*M* = 23.87, *SD* = 3.86). Most of the students (62.5%) did not indicate their faculty, but those who did belonged to the following faculties: mathematics and natural sciences (16.8%), law and economics (8.7%), philosophy (7.2%), medicine (3.4%), and theology (0.5%).

### Impact of the situational and application-related characteristics

As a first step, *random intercept only models* (RIO) with no explanatory variables were calculated for each group (see Additional file 3: Table S3). The RIO model, which estimates the average intention to use a self-test across all vignettes and respondents, was the lowest for the ST group (b_ST_ = 51.51), higher for the HPH group (b_HPH_ = 65.82), and the highest for the HPD condition (b_HPD_ = 70.23). The *error variance between* amounted to δ_ST_ = 218.56, δ_HPH_ = 302.96, and δ_HPD_ = 217.04 in the ST, HPH, and HPD groups, respectively. The *error variance within* amounted to ε_ST_ = 747.95, ε_HPH_ = 515.65, and ε_HPD_ = 463.74, respectively. Hence, about 77.4%, 63.0%, and 68.1% of the total variance of the respective ST, HPH, and HPD groups was within-person variance, leaving ample room for including predictors.

The second step was to investigate the predictive value of the situational and application-related characteristics on the intention to test, by calculating *random intercept models* with all vignette factors (RI_V_all_). This resulted in a better model fit for the HPH and HPD groups compared to the empty models (χ^2^_HPH_ = 40.54, p_HPH_ < 0.01; χ^2^_HPD_ = 35.23, p_HPD_ < 0.05), but this was not the case for the ST group (χ_ST_^2^ = 14.92, *p* = 0.78, see Table 2). However, for all three groups, the deviances and the *error variances within* were lower in the model with all vignette factors compared to the empty model. Accordingly, in the ST group, the *error variance within* declined from 747.95 in the RIO model to 672.58 in the RI_V_all_ model. This means that about 10.1% of the *error variance within* could be explained by adding the situational and application-related predictors to the empty model. For the HPH 10.6% and for the HPD group 11.2% of the *error variance within* were explained by the vignette factors.

**Table 2:** Multilevel model with vignette characteristics and the criterion “intention to use a test” separately for the groups ST, HPH, and HPD

	RI_V_all_					
	ST		HPH		HPD	
Fixed effects	b	(SE)	b	(SE)	b	(SE)
intercept	36.00***	(7.03)	43.68***	(5.08)	55.24***	(4.48)
Vignette characteristics						
Application purpose						
Risk assessment^RefA^						
Clinical diagnostics	0.68	(6.31)	6.29	(3.54)	5.86	(5.08)
Drug effect	1.08	(6.50)	8.53*	(3.67)	9.58*	(4.64)
Early detection of a disease	−1.57	(6.65)	8.62*	(4.27)	13.22***	(3.86)
Monitoring	6.40	(6.47)	7.93	(4.19)	11.78**	(4.20)
Therapy diagnostics	6.50	(7.74)	8.98*	(3.89)	5.86	(5.08)
Seriousness of the situation						
Acute and life-threatening^RefB^						
Acute, but not life-threatening	6.66	(5.37)	2.96	(3.97)	−4.08	(4.11)
Chronic, but not life-threatening	6.67	(5.60)	3.44	(3.87)	−0.096	(3.59)
Chronic, slowly advancing and life-threatening	2.30	(5.51)	6.19	(4.30)	2.00	(3.99)
Analysis and feedback						
Transmitted automatically and the result is communicated in written form^RefC^						
Transmitted automatically and only a conspicuous result is communicated by a health professional	^RefC^-1.57	(5.77)				
Transmitted automatically and the result is communicated by a health professional	^RefC^1.40	(5.45)				
Analyzed in a laboratory and only a conspicuous result is communicated by a health professional^RefD^						
Analyzed automatically, and the result is displayed immediately	^RefC^-5.41	(5.27)	^RefD^4.58	(3.28)	^RefD^3.46	(3.44)
Analyzed in a laboratory and the result is communicated in written form			^RefD^4.31	(3.34)	^RefD^-1.75	(3.25)
Analyzed in a laboratory and the result is communicated by a health professional			^RefD^6.21	(3.53)	^RefD^5.68	(3.55)
Medical expertise of the tested person						
No^RefE^						
Unprofessional	4.42	(4.94)	0.96	(3.16)	1.09	(3.10)
Professional	13.33**	(4.96)	9.80**	(3.12)	8.66**	(3.02)
Emotional support						
Not present^RefF^						
Potentially available	7.93	(4.60)	5.25	(3.27)	3.52	(3.18)
Personally present	8.64	(4.87)	9.71**	(3.14)	1.76	(3.66)
Random effects						
δ_im_ (error variance between)	268.22		303.78		227.83	
ε_ij_ (error variance within)	672.58		461.16		411.73	
Deviance	3247.86		4468.87		3985.02	
N_O_ / N_G_	337/183		478/196		433/192	

N_o_ = Number of observations/vignettes

N_G_ = Number of groups/respondents

* *p* < 0.05, ** *p* < 0.01, *** *p* < 0.001

*Seriousness of the situation* and *analysis and feedback of the test results* did not affect the intention to self-test for any group. Self-test use was significantly predicted by only one vignette factor: *medical expertise of the tested person*. The intention to use a self-test was on average 13.33 points higher for participants who imagined to have the professional knowledge to evaluate the test results, compared to no knowledge (on a scale from 1 to 100). A professional knowledge compared to no knowledge also significantly increased the intention to be tested by a health professional at home or at a doctor’s office (b_HPH_ = 9.80, b_HPD_ = 8.66), but the impact of the *medical expertise of the tested person* was the highest for the ST group. While the vignette factor *application purpose* did not significantly influence the intention to use a self-test, it had a significant effect on the HPH and HPD groups, which stated a higher intention to be tested when the *application purpose* was drug effect or early detection of a disease compared to risk assessment. Additionally, the intention to test was significantly increased for the *application purpose* monitoring for the HPD group and therapy diagnostics for the HPH group compared to risk assessment. The presence of *emotional support* affected only the HPH group, whose intention to test was on average 9.71 higher if a closely related person who could provide emotional support was present, compared to the absence of such a person.

### Impact of the psychological characteristics

In the third step, a model with *technological affinity* added as a predictor (RI_V_all__P_TA_) was calculated (Table 3). The addition of this predictor resulted in a better model fit than the RI_V_all_ model for all three settings (χ^2^_ST_ = 13.43, p_ST_ < 0.01; χ^2^_HPH_ = 9.33, p_HPH_ < 0.05; χ^2^_HPD_ = 10.58, p_HPD_ < 0.05). Additionally, the *error variance between* declined in the ST group from 268.22 to 216.28; thus, 19.4% of this variance was explained by adding *technological affinity* to the RI_V_all_ model (compared to 8.3% *error variance between* of the HPH group and 6.1% of the HPD group). The results of the RI_V_all__P_TA_ model showed that, while the intention to use a self-test significantly increased with higher values on the *technological affinity enthusiasm* scale (b_ST_ = 6.21), the intention of being tested by a health professional at a doctor’s office/hospital significantly increased with higher values on the *technological affinity positive attitude* scale (b_HPD_ = 7.29). However, the impact of the situational and application-related characteristics on the intention to test did not change when adding the *technological affinity* scales to the model.

**Table 3:** Multilevel models with vignette and respondent characteristics and the criterion “intention to use a test” separately for the groups ST, HPH, and HPD

	RI_V_all__P__TA_						RI_V_all__P_all_					
	ST		HPH		HPD		ST		HPH		HPD	
Fixed effects	b	(SE)	b	(SE)	b	(SE)	b	(SE)	b	(SE)	b	(SE)
intercept	36.65***	(6.94)	43.69***	(5.05)	55.87***	(4.44)	48.71***	4.65	52.40***	(3.91)	59.42***	(3.35)
Vignette characteristics												
Application purpose												
Risk assessment^RefA^												
Clinical diagnostics	−0.30	(6.24)	6.41	(3.53)	5.10	(5.05)	2.10	(4.17)	3.90	(2.70)	−2.21	(3.80)
Drug effect	0.91	(6.42)	8.57*	(3.66)	9.12*	(4.60)	−1.29	(4.27)	2.69	(2.82)	2.05	(3.48)
Early detection of a disease	−2.50	(6.59)	8.72*	(4.26)	12.64**	(3.85)	2.16	(4.38)	5.93	(3.25)	4.61	(2.91)
Monitoring	5.67	(6.39)	8.25*	(4.17)	11.27**	(4.16)	6.16	(4.28)	2.28	(3.21)	3.98	(3.15)
Therapy diagnostics	5.94	(7.66)	9.31*	(3.88)	10.32**	(3.66)	−0.41	(5.10)	4.85	(2.98)	0.59	(2.79)
Seriousness of the situation												
Acute and life-threatening^RefB^												
Acute, but not life-threatening	8.25	(5.33)	2.46	(3.95)	−3.16	(4.08)	1.02	(3.56)	−1.53	(3.05)	0.83	(3.09)
Chronic, but not life-threatening	7.91	(5.53)	3.49	(3.85)	0.129	(3.56)	6.46	(3.74)	1.73	(2.97)	2.63	(2.70)
Chronic, slowly advancing and life-threatening	2.90	(5.48)	5.88	(4.28)	1.80	(3.96)	1.59	(3.64)	6.08	(3.27)	3.94	(2.96)
Analysis and feedback												
Transmitted automatically and the result is communicated in written form^RefC^												
Transmitted automatically and only a conspicuous result is communicated by a health professional	^RefC^-3.44	(5.72)					^RefC^-0.44	(3.82)				
Transmitted automatically and the result is communicated by a health professional	^RefC^0.23	(5.37)					^RefC^0.93	(3.60)				
Analyzed in a laboratory and only a conspicuous result is communicated by a health professional^RefD^												
Analyzed automatically, and the result is displayed immediately	^RefC^-5.94	(5.20)	^RefD^5.02	(3.27)	^RefD^3.11	(3.41)	^RefC^-0.38	(3.46)	^RefD^5.16*	(2.49)	^RefD^0.96	(2.54)
Analyzed in a laboratory and the result is communicated in written form			^RefD^4.62	(3.33)	^RefD^-1.80	(3.22)			^RefD^3.87	(2.54)	^RefD^-1.75	(2.41)
Analyzed in a laboratory and the result is communicated by a health professional			^RefD^6.17	(3.53)	^RefD^6.41	(3.53)			^RefD^1.90	(2.71)	^RefD^5.30*	(2.64)
Medical expertise of the tested person												
No^RefE^												
Unprofessional	5.06	(4.88)	0.78	(3.15)	1.37	(3.07)	2.39	(3.24)	1.18	(2.41)	−0.85	(2.30)
Professional	13.80**	(4.89)	9.43**	(3.11)	8.36**	(2.99)	7.17*	(3.27)	7.04**	(2.38)	2.92	(2.26)
Emotional support												
Not present^RefF^												
Potentially available	7.46	(4.56)	5.44	(3.27)	2.72	(3.16)	3.76	(3.05)	2.54	(2.50)	4.11	(2.36)
Personally present	8.61	(4.81)	9.66**	(3.13)	0.94	(3.63)	3.78	(3.19)	4.41	(2.41)	4.70	(2.72)
Respondent characteristics												
Technology affinity enthusiasm	6.21*	(2.90)	2.14	(2.50)	2.41	(2.35)	2.07	(2.02)	0.54	(1.92)	3.24	(1.71)
Technology affinity competence	0.16	(3.20)	2.29	(2.97)	−1.02	(2.64)	2.07	(2.26)	2.87	(2.31)	−2.50	(1.95)
Technology affinity positive attitude	4.04	(3.60)	4.79	(3.01)	7.29*	(3.01)	0.17	(2.48)	2.11	(2.31)	−0.43	(2.22)
Perceived susceptibility							0.24	(0.34)	0.37	(0.33)	0.40	(0.31)
Self-efficacy							−0.04	(0.31)	−0.51	(0.31)	0.07	(0.27)
Perceived severity							0.16***	(0.04)	0.23***	(0.04)	0.19***	(0.03)
Outcome expectancy							0.89***	(0.05)	0.68***	(0.04)	0.69***	(0.04)
Random effects												
δ_im_ (error variance between)	216.28		278.52		213.94		113.18		162.74		101.95	
ε_ij_ (error variance within)	676.11		461.75		406.46		286.40		267.99		230.54	
Deviance	3234.43		4459.54		3974.44		2959.49		4200.21		3712.40	
N_O_ / N_G_	337/183		478/196		433/192		337/183		478/196		433/192	

*N*_*o*_ Number of observations/vignettes

*N*_*G*_ Number of groups/respondents

* *p* < 0.05, ** *p* < 0.01, *** *p* < 0.001

Finally, in the fourth model, the health psychological factors were added as predictors (RI_V_all__P_all_, see Table 3). This addition resulted in a better model fit than the RI_V_all__P_TA_ model for all three settings (χ^2^_ST_ = 274.94, p_ST_ < 0.001; χ^2^_HPH_ = 259.33, p_HPH_ < 0.001; χ^2^_HPD_ = 262.05, p_HPD_ < 0.001). The *error variance within* declined from the RI_V_all__P_TA_ to the RI_V_all__P_all_ model in all three test settings. About 47.7%, 41.57% and 52.35% of the *error variance within* in the ST, HPH and HPD groups, respectively, could be explained by adding the health psychological predictors to the RI_V_all__P_TA_ model.

*Perceived severity* (b_ST_ = 0.16, b_HPH_ = 0.23, b_HPD_ = 0.19) and *outcome expectancy* (b_ST_ = 0.89, b_HPH_ = 0.68, b_HPD_ = 0.69) significantly predicted the intention to test for all three test settings. Furthermore, in the RI_V_all__P_all_ model, the impact of *technological affinity* and the vignette factor *application purpose* disappeared, whereas the vignette factor *analysis and feedback of the test results* had a significant impact on the HPH and HPD groups. However, the professional expertise of the tested person remained a significant predictor of the intention to self-test (b_ST_ = 7.17) and being tested by a health professional at home (b_HPH_ = 7.04).

## Discussion

### Main findings and comparison with other studies

This study had three objectives. First, the impact of situational and application-related characteristics of the test situation on the intention to use a self-test (ST) versus being tested by a health professional at home (HPH), or in a doctor’s office/hospital (HPD), were experimentally investigated. A factorial survey was conducted enabling the systematic variation of a set of situational and application-related characteristics. The results suggested that the intention to use a self-test was only predicted by the *medical expertise of the tested person*. Exclusively participants who were asked to imagine themselves as having the professional knowledge to evaluate the test results had a higher intention to use a self-test than those who had no medical knowledge. Professional medical expertise was also important, though to a lesser degree, in the decision of being tested by a health professional at home or at a doctor’s office. Presumably, participants did not think that it made sense to conduct a test by themselves or by a health professional if they do not understand the test results. While the *seriousness of the situation* and the *analysis and feedback of the test results* did not predict any intention to test, the *application purpose* did influence the decision whether to be tested by a health professional at home or at a doctor’s office. Interestingly, the presence or absence of a closely related person who could provide *emotional support* did not affect the intention to self-test, but the presence of a supportive person did raise the probability of the intention of being tested by a health professional at home.

The second objective of this study was to investigate whether the core concepts of health behaviour theories can predict the intention to use a self-test. The results showed that there was no significant association between *perceived susceptibility* and the intention to self-test, to be tested at home, or at a doctor’s office. However, previous research on the psychological determinants of self-testing for cholesterol, glucose and HIV in a cross-sectional survey has found that *perceived susceptibility* was a significant predictor of the use of cholesterol and HIV self-tests, but not of glucose [3]. The relationship between *perceived susceptibility* and the intention to use a self-test therefore seems to depend on the specific health risk or disease under investigation. Since our factorial survey did not specify the health risk or disease, but instead focussed on the participants’ views on the chance of contracting a non-specific ‘acute vs. chronic, non-life-threatening vs. life-threatening’ disease, the association between *perceived susceptibility* and the intention to test did not become apparent.

Although in previous research, *self-efficacy* has been shown to be an important predictor of the intention to attend and actual attendance of screening programmes, as well as of self-testing for cholesterol, glucose and HIV [3, 20, 40, 41], in our factorial survey no such associations were found. However, in our survey *self-efficacy* was assessed with the GSE, whereas in previous research the items to assess self-efficacy were specifically related to self-testing [3]. A question is whether standardized instruments should be adopted for the specific health behaviour.

In comparison, results for *perceived severity* and *outcome expectancy* were in accordance with the theoretical predictions, because both significantly predicted the intention to be self-tested and tested by a health professional at home or at a doctor’s office. In contrast, Grispen et al. [3] found no association between *perceived severity* and self-testing for glucose, HIV or cholesterol. According to Hahn and Lengerke [18], *outcome expectancy* is equivalent to perceived barriers and perceived benefits from the HBM or the response efficacy from the PMT. While perceived benefits significantly predicted the use of all three self-tests, test-specific associations were identified for response efficacy and perceived barriers [3].

The third objective was to investigate whether the predictive value of the core concepts of health behaviour theories can be improved by adding *technological affinity*. The results showed that the addition of *technological affinity* to the situational and application-related factors significantly predicted the intention to self-test, which supported our assumption about the positive relationship between *technological affinity* and self-testing to some degree. Additionally, the hypothesised benefit of assessing *technological affinity* with different subscales was confirmed, because the intention to use a self-test was only predicted by a higher technical enthusiasm, whereas people who assigned themselves no technological competence, but still had a positive attitude towards technology, preferred to be tested at a doctor’s office. However, when adding the health psychological predictors to the final model, *technological affinity* turned out to be statistically non-significant. This suggests that the health psychological predictors incorporated and superseded the predictive value of *technological affinity*.

### Strengths and limitations

*Self-efficacy* was measured with the GSE scale, which enabled the comparison of the results with those of other studies. However, a phrasing in terms of the individual’s confidence in one’s capability to successfully perform a self-test would have been more sensitive and in line with the theoretical assumptions. Second, as *perceived susceptibility* was adopted to fit to the vignette factor ‘seriousness of a situation’, this study has investigated the *perceived susceptibility* of getting a non-specific ‘acute vs. chronic, non-life-threatening vs. life-threatening’ disease. In future studies, however, the individual’s belief of the chance of contracting *a certain disease/condition* should also be investigated, since significant associations might depend on the specification of the disease/condition to be tested. Third, a factorial survey was chosen because it allows an *experimental* investigation of the impact of situational and application-related factors. However, the display of fictive scenarios might be an additional reason for why there was no or only little association between *self-efficacy* and *perceived susceptibility* with the intention to test. The results may also have had a low external validity, but they are distinguished by a high internal validity. Fifth, order effects cannot be excluded. This study aimed at a balanced ratio of the six values of the factor *application purpose*, so that 30 vignettes of each of its values were drawn randomly without replacement. Sixth, the sample consisted only of university students, who, compared to the general population, may have specific characteristics such as a higher education level or a higher family income. On the other hand, a homogenous sample is advantageous for experimental investigations, because they are less biased. Consequently, future work should re-examine the research questions posed here by comparing actual self-testers with non-self-testers in the general population.

## Conclusions

Despite the abovementioned limitations of this study, it can be concluded that the situational and application-related determinants which predicted the intention to use a self-test differed from those predicting the intention of being tested by a health professional at home or in a doctor’s office/hospital. In fact, the only situational and application-related factor which predicted the intention to self-test was a professional medical expertise of the tested person. Although the most frequently stated advantages of self-testing include the faster diagnostics and higher privacy protection [14], situational and application-related factors such as ‘analysed automatically, and the result is displayed immediately’ did not significantly predict the intention to use a self-test. Additionally, *technological affinity* predicted the intention to self-test, but when the core concepts of social-cognitive health behaviour theories were added, the impact of *technological affinity* was incorporated. Therefore, it can be concluded that the existing social-cognitive health behaviour theories can be applied to predict the intention to use a self-test and do not need to be extended by *technological affinity*. However, since vignettes were used to investigate the determinants of the *intention* to use a self-test, additional studies comparing *actual* self-testers with non-self-testers are necessary to fully understand the psychological, situational and application-related determinants of self-test use.

## Additional files

Additional file 1: Table S1. Overview of psychological constructs, conceptual definitions, items, and answering options. Description: This file contains the additional Table 1 which gives an overview of the psychological constructs, conceptual definitions, items, and answering options of the survey. (DOC 69 kb)

Additional file 2: Table S2. Descriptive statistics of the vignette characteristics in dependence of the criterion “intention to test” separately for the groups ST, HPH, and HPD. Description: This file contains the additional Table 2 which gives an overview of the descriptive statistics of the vignette characteristics in dependence of the criterion “intention to test” separately for the groups ST, HPH, and HPD. (DOC 91 kb)

Additional file 3: Table S3. Random intercept only model for the criterion “intention to use a test” separately for the groups ST, HPH, and HPD”. Description: This file contains the additional Table 3 which shows the results of the random intercept only model for the criterion intention to use a test separately for the groups ST, HPH, and HPD. (DOC 69 kb)

Additional file 4: Data_Online Survey_Determinants of intention to self-test.SAV”. Description: The file contains the data of the survey. (SAV 79 kb)

## Abbreviations

BRAHMS: Berlin Risk Appraisal and Health Motivation Study
GSE: General Self-Efficacy scale
HAPA: Health Action Process Approach
HBM: Health Belief Model
HPD: Health professional at a doctor’s office
HPH: Health professional at home
LOCs: Lab-on-a-chip systems
PMT: Protection Motivation Theory
RI_V_all_: Random intercept models with all vignette factors
RI_V_all__P_all_: Random intercept models with all vignette factors and all psychological factors
RI_V_all__P_TA_: Random intercept models with all vignette factors and technological affinity
RIO: Random intercept only models
ST: Self-test
TA-EG: German Technological Affinity Assessment
TPB: Theory of Planned Behavior
