# Supplementary material for: Psychological, situational and application-related determinants of the intention to self-test: a factorial survey among students
Source: BMC Health Serv Res. 2017 Jul 10;17:468. doi: 10.1186/s12913-017-2394-x (PMC5504798; doi:10.1186/s12913-017-2394-x)
Supplement: Supplementary file 1 — Overview of psychological constructs, conceptual definitions, items, and answering options. Description: This file contains the additional Table 1 which gives an overview of the psychological constructs, conceptual definitions, items, and answering options of the survey. (DOC 69 kb) [file 12913_2017_2394_MOESM1_ESM.doc]

## Additional file - Table S1 - Overview of psychological constructs, conceptual definitions, items, and answering options

| **Perceived susceptibility:** the individual´s belief of the chance of contracting a certain disease/condition |
| --- |
| - How high do you rate the probability that sometime you will get ...   - an acute and life-threatening disease?   - an acute, but not life-threatening disease?   - a chronic, slowly progressing, and life-threatening disease?   - a chronic, but not life-threatening disease?   - Response format: Likert scale from 1 = very unlikely to 5 = very likely |
| **Perceived severity:** the individual´s belief of the seriousness of a certain disease/condition |
| - Now, please imagine there would be a corresponding risk of contracting a disease to you personally. How serious would it be for your health, if the test depicted in the above situation would not be conducted? - Response format: visual analogue scale from 1 = not severe to 100 = very severe |
| **Outcome expectancy:** the individual´s weighting of the positive and negative consequences of acting and not acting |
| - What would be the consequences for your personal health, if you would conduct a test as depicted in the above situation? - Response format: visual analogue scale from 1 = very negative to 100 = very positive |
| **Self-efficacy:** the individual´s confidence in one´s capability to successfully perform a certain action |
| - - The General Self-Efficacy Scale (GSE) by Schwarzer & Jerusalem (1995)   - Ten items, e.g. “I can always manage to solve difficult problems if I try hard enough.”   - Response format: Likert scale from 1 = not at all true, 2 = hardly true, 3 = moderately true, 4 = exactly true |
| **Technological affinity:** personality trait which manifests in a positive attitude, enthusiasm, and trust in electronic devices |
| - - Technological affinity questionnaire (TA-EG) by Karrer, Glaser, Clemens, and Bruder   - Enthusiasm five items, e.g. “I am excited, when a new electronic device comes on the market.”   - Competence four items, e.g. “I know most of the functions of the electronic devices that I own.”   - Positive attitudes five items, e.g. “Electronic devices enable a high standard of living.”   - Response format: Likert scale from 1 = not at all true to 5 = exactly true |
| **Intention to use a test:** the individual´s intention of using a test as described in a fictive scenario |
| - - Would you make use of a test that is conducted as described in the situation above?   - Response format: visual analogue scale from 1 = certainly not to 100 = most certainly |
