# Supplementary material for: Psychological, situational and application-related determinants of the intention to self-test: a factorial survey among students
Source: BMC Health Serv Res. 2017 Jul 10;17:468. doi: 10.1186/s12913-017-2394-x (PMC5504798; doi:10.1186/s12913-017-2394-x)
Supplement: Supplementary file 2 — Descriptive statistics of the vignette characteristics in dependence of the criterion “intention to test” separately for the groups ST, HPH, and HPD. Description: This file contains the additional Table 2 which gives an overview of the descriptive statistics of the vignette characteristics in dependence of the criterion “intention to test” separately for the groups ST, HPH, and HPD. (DOC 91 kb) [file 12913_2017_2394_MOESM2_ESM.doc]

**Additional file - Table S2 - Descriptive statistics of the vignette characteristics in dependence of the criterion „intention to test“ separately for the groups ST, HPH, and HPD**

|  | | **ST** | | **HPH** | | **HPD** | | **Total** | |
| --- | --- | --- | --- | --- | --- | --- | --- | --- | --- |
|  | | M | SD | M | SD | M | SD | M | SD |
| **Application purpose** | |  |  |  |  |  |  |  |  |
|  | Risk assessment | 50.40 | 27.81 | 58.97 | 30.14 | 61.12 | 25.86 | 57.89 | 28.49 |
|  | Clinical diagnostics | 49.94 | 31.11 | 65.75 | 26.81 | 66.62 | 26.72 | 60.91 | 29.08 |
|  | Drug effect | 54.79 | 30.59 | 68.38 | 26.39 | 70.88 | 23.55 | 63.70 | 28.28 |
|  | Early detection of a disease | 49.47 | 33.44 | 66.82 | 31.95 | 71.59 | 24.23 | 64.04 | 30.72 |
|  | Monitoring | 53.14 | 33.03 | 67.15 | 26.16 | 73.00 | 25.05 | 65.60 | 28.78 |
|  | Therapy diagnostics | 54.00 | 30.96 | 67.33 | 30.77 | 72.42 | 28.31 | 67.79 | 30.12 |
| **Seriousness of the situation** | |  |  |  |  |  |  |  |  |
|  | acute and life-threatening | 51.84 | 31.92 | 63.30 | 31.58 | 69.81 | 25.34 | 61.42 | 30.51 |
|  | acute, but not life-threatening | 52.46 | 27.97 | 64.41 | 28.06 | 67.39 | 27.18 | 62.63 | 28.25 |
|  | chronic, but not life-threatening | 54.12 | 31.48 | 66.12 | 28.54 | 69.75 | 26.68 | 64.51 | 29.21 |
|  | chronic, slowly advancing and life-threatening | 48.58 | 33.12 | 67.23 | 27.53 | 72.85 | 24.84 | 64.72 | 29.56 |
| **Analysis and feedback** | |  |  |  |  |  |  |  |  |
|  | transmitted automatically and the result is communicated in written form | 52.11 | 29.714 | - | - | - | - | 52.11 | 29.71 |
|  | transmitted automatically and only a conspicuous result is communicated by a health professional | 52.89 | 30.36 | - | - | - | - | 52.89 | 30.36 |
|  | transmitted automatically and the result is communicated by a health professional | 54.07 | 31.91 | - | - | - | - | 54.07 | 31.91 |
|  | analysed in a laboratory and only a conspicuous result is communicated by a health professional | - | - | 59.54 | 29.16 | 67.48 | 23.54 | 63.02 | 27.09 |
|  | analysed automatically, and the result is displayed immediately | 46.18 | 32.62 | 67.06 | 26.11 | 71.15 | 25.79 | 63.98 | 29.08 |
|  | analysed in a laboratory and the result is communicated in written form | - | - | 67.72 | 30.38 | 67.35 | 29.52 | 67.51 | 29.84 |
|  | analysed in a laboratory and the result is communicated by a health professional | - | - | 70.62 | 27.60 | 76.10 | 23.61 | 73.02 | 26.00 |
| **Medical expertise of the tested person** | |  |  |  |  |  |  |  |  |
|  | no | 49.91 | 31.41 | 59.55 | 31.89 | 66.61 | 26.88 | 59.46 | 30.61 |
|  | unprofessional | 51.01 | 32.04 | 63.11 | 27.87 | 69.08 | 26.37 | 61.13 | 29.65 |
|  | professional | 56.18 | 29.38 | 71.34 | 26.08 | 73.63 | 24.63 | 68.99 | 27.08 |
| **Emotional support** | |  |  |  |  |  |  |  |  |
|  | not present | 49.83 | 29.75 | 62.08 | 29.57 | 68.33 | 26.18 | 61.11 | 29.26 |
|  | potentially available | 52.97 | 31.83 | 66.44 | 27.53 | 71.53 | 26.62 | 64.09 | 29.51 |
|  | personally present | 53.58 | 32.15 | 67.06 | 28.87 | 70.17 | 25.40 | 64.89 | 29.28 |

Note^a^: independently at home without the presence of a health professional (ST)

Note^b^: at home, conducted by a health professional (HPH)

Note^c^: in the doctor’s office/hospital by a health professional (HPD)
