# Supplementary material for: Psychological, situational and application-related determinants of the intention to self-test: a factorial survey among students
Source: BMC Health Serv Res. 2017 Jul 10;17:468. doi: 10.1186/s12913-017-2394-x (PMC5504798; doi:10.1186/s12913-017-2394-x)
Supplement: Supplementary file 3 — Random intercept only model for the criterion “intention to use a test” separately for the groups ST, HPH, and HPD”. Description: This file contains the additional Table 3 which shows the results of the random intercept only model for the criterion intention to use a test separately for the groups ST, HPH, and HPD. (DOC 69 kb) [file 12913_2017_2394_MOESM3_ESM.doc]

**Additional file - Table S3 - Random intercept only model for the criterion “intention to use a test“ separately for the groups ST, HPH,** and HPD

|  |  |  | **RIO** | | | | | | | | |
| --- | --- | --- | --- | --- | --- | --- | --- | --- | --- | --- | --- |
|  |  |  | **ST** | | | **HPH** | | | **HPD** | | |
| **Fixed effects** | | | b |  | (SE) | b |  | (SE) | b |  | (SE) |
|  | intercept | | 51.51 | *** | (1.89) | 65.82 | *** | (1.66) | 70.23 | *** | (1.52) |
| **Random effects** | | |  |  |  |  |  |  |  |  |  |
|  | δ_im_ (error variance between) | | 218.56 |  |  | 302.96 |  |  | 217.04 |  |  |
|  | ε_ij_ (error variance within) | | 747.95 |  |  | 515.65 |  |  | 463.74 |  |  |
|  | Deviance | | 3262.78 |  |  | 4509.40 |  |  | 4020.24 |  |  |
|  | N_O_ / N_G_ | | 337/183 |  |  | 478/196 |  |  | 433/192 |  |  |

Note^a^: independently at home without the presence of a health professional (ST)

Note^b^: at home, conducted by a health professional (HPH)

Note^c^: in the doctor’s office/hospital by a health professional (HPD)

N_o_ = Number of observations/vignettes

N_G_ = Number of groups/respondents

* p<0.05, ** p<0.01, *** p<0.001
